# Putative Natural History of CoViD-19

**DOI:** 10.6026/97320630016398

**Published:** 2020-05-31

**Authors:** Francesco Chiappell

**Affiliations:** 1 Professor Emeritus, UCLA Center for the Health Sciences, Los Angeles, CA

**Keywords:** Corona Virus Disease 2019 (CoViD-19), Severe Acute Respiratory Syndrome Corona Virus2 (SARS-CoV2), Exopeptidase CD26, Peptidase Targeted Immunoregulation (PeTIr), angiotensin-converting enzyme-2 (ACE2), transmembrane protease serine-2 (TMPRSS2), Basigin CD147, clustered regularly interspaced short palindromic repeats (CRISPR), transferrin receptor CD71, platelet tissue factor CD142, cytokine synthesis inhibitory factor (IL10)

## Abstract

The Severe Acute Respiratory Syndrome Corona Virus2 (SARS-CoV2) is responsible for Corona Virus Disease 2019 (CoViD-19), the pandemic that has afflicted close to two million people worldwide, and has taken the lives of over 120,000 patients since its first report in late December 2019. Per million people globally, the infection rate is close to 250 with a death rate of close to 14 (death rate average global death rate: 6.06%; for comparison, revised estimate of the 1918 influenza pandemic had an average global death rate of 5.4% [1]). About 400,000 SARS-CoV2-positive patients have been declared 'recovered', although it is not clear to date what exactly that entails. To be clear, the natural history of SARS-CoV2 infection and of the patho-physiology of CoViD-19 remains shrouded in relative confusion, in part due to the exceedingly virulent nature of the virus, as manifest by its elevated morbidity and mortality, and the fast accumulation of clinical observations and research evidence. Many pieces of a complex puzzle are emerging all at once and their organization into a coherent and cogent picture of the natural history of CoViD-19 is arduous and still wanting. Here, we discuss the recent findings in the context of the available evidence. We propose a putative prediction model of the natural history of CoViD-19. We highlight putative loci and modes of therapeutic intervention that may become beneficial preventive and treatment modalities for individuals at risk of SARS-CoV2 infection and CoViD-19 patients.

## Background

The virus responsible for CoViD-19 is a positive-sense single-stranded RNA (+ssRNA) virus, the second virus of the Corona family to induce symptoms of severe acute respiratory syndrome (SARS-Cov2). It possesses a single linear RNA segment of 29,903 bases (NCBI genome ID: MN908947), which code for four structural proteins: S (spike), E (envelope), M (membrane), and N (nucleocapsid), and other minor proteins. N encapsulates the viral genome, and S, E, and M play a specific role in the viral inflammasome: M proffers much of the viral morphogenesis; while E, despite it's relatively small size, dramatically influences virus replication and pathogenicity. E critically mediates viral budding, assembly, intracellular trafficking, and consequently overall virulence. E's transmembrane domain harbors an ion channel activity, and sequences within the middle region of its carboxy-terminus and its most carboxy-terminal end that can act as a PDZ domain and anchor receptor proteins in the membrane to cytoskeletal components. Consequently, E elicits much of the inflammation by the virus, including in the lung parenchyma and pulmonary alveoli. S is composed of two subunits that are shielded and camouflaged from immune recognition as nonself by glycosylation. Glycoprotein S subunit 1 (S1) binds to and blunts the expopeptidase activity of cluster of differentiaton-26 (CD26) (i.e., dipeptidyl peptidase-4, aka adenosine deaminase complexing protein-2) [2]. We [3] and others [4] investigated and characterized the immunoregulatory role of CD26 in cleaving and inactivating a broad range of substrates, from growth factors and cytokines to neuro-peptides and vasoactive peptides. Indeed, CD26 is an important member of the Peptidase Targeted Immuno-regulation (PeTIr) membrane-associated enzyme family [5]. Taken together, these lines of evidence suggest as strong a potential role for CD26 as a molecular target for novel treatment modalities in T-cell lymphoid malignancies [6] as possibly in CoViD-19.

S attaches to the host cell membrane via the single-spanning transmembrane Zinc-dependent angiotensin-converting enzyme-2 (ACE2), ubiquitously expressed in most organ cells, from lung alveolar epithelial cells to small intestine enterocytes, vascular endothelial cells as well as cerebral and neural tissue. ACE2 plays an essential regulatory role in the renin-angiotensin system, which maintains homeostasis and protects a variety of organs, including the heart, kidneys and lungs, from the damaging effects of hypertension, diabetes, and cardiovascular disease [7,9]. To fuse to the host membrane following attachment, S must be primed by transmembrane protease serine-2 (TMPRSS2), which clips S to expose the viral fusion peptide that permits release of the viral RNA into the cell [8]. S is also endowed with a furin-like cleavage site [10], remarkably similar to one of the proteases responsible for the proteolytic cleavage of HIV envelope polyprotein precursor gp160 to gp120 and gp41 prior to viral assembly. In brief, anti-S vaccines or S-competitors for ACE2 binding, or protease inhibitors that block either TMPRSS2 or furin activity could all strong promise for preventing SARS-CoV2 infection [8-10]. S can seemingly also bind to the ubiquitous membrane-bound Ig-superfamily metallo-protease inducer, basigin (CD147) to invade the host cell [11]. Thus, meplazumab, a humanized anti-CD147 antibody, currently being tested with CoViD-19 patients [12], is likely to show success. CD147 is an essential receptor for erythrocyte invasion by Plasmodium falciparum, the most virulent of the parasites that cause malaria. Hydroxychloroquine, used against the malarial parasites for over three decades [13], may interfere with the same pathway of SARS-Cov2 host cell invasion. But not even the most recent systematic review can conclusively establish the efficacy of any chloroquine derivatives in patients with CoViD-19, beyond limiting the replication of SARS-CoV2 in vitro [14].

To be clear, hydroxychloroquine (aka, Plaquenil) easily penetrates the plasma membrane and, because of its basic nature, increases the pH of lysosomes, thus blunting the metabolic processing of foreign pathogens and leading to a plethora of cellular consequences, not the least of which being, in antigen-presenting cells, a significant block of toll-like receptors, and consequential reduction in antigen-presenting cell activation and the inflammatory process. Hyroxychloroquine is therefore widely used for autoimmune diseases such as systemic lupus erythematosus and rheumatoid arthritis [15]. By this rationale, hydroxychloroquine is expected to contain the cytokine storm in CoViD-19, against any confirmatory evidence at this time. Once on the inside, the 5' methylated cap and 3' polyadenylated tail allows the positive-sense RNA viral genome to be directly translated by the host cell's ribosome. The viral protein and nucleic acid components are reassembled, and multiple virion particles released from the infected cell by shedding.

The genome has several overlapping open reading frames, which encode for the viral replicase/transcriptase polyprotein, and the major structural viral proteins, S, E, M and N. Each site can be targeted by tailor-made anti-virals crafted on the model of the Corona viruses that have infected human populations [16]. Case in point, FDA-approved anti-parasitic ivermectin that inhibits the replication of SARS-CoV2 in vitro by over 5,000 fold in 48 h [17], imatinib and related Abl-kinase inhibitors [18,19], and others adjunctive pharmacologic therapy (20) including early use of oseltamivir (tamiflu), a neuraminidase inhibitor that successfully interferes with the process of viral shedding and reduces mortality in critically ill patients with influenza virus [21] may be successful in CoViD-19. The complexity of the SARS-CoV2 and of resulting CoVid-19 suggests that antiviral therapy must be aided by targeted immunotherapy [22] and other selected molecular tools directed at controlling and regulating the overwhelming inflammatory response. We predict that clustered regularly interspaced short palindromic repeats (CRISPR) protocols may be helpful in forging a better understanding of the natural history of the pathology of CoVid-19, and in developing novel targeted anti-SARS-CoV2 therapies.

## Methodology:

CRISPR gene editing is a conceptually relatively simple protocol that involves delivering the Cas9 nuclease, a bacterial a dual RNA-guided DNA endonuclease enzyme, complexed with a synthetic guide RNA (gRNA) into a cell to activate specific cuts within the host cell's genome, and allowing existing genes to be removed or new ones added in situ and in vivo. By this genetic engineering technique, the genomes of living organisms may be modified, or infecting viral genomes can be rapidly and reliably detected and counted of virus for diagnostic and prognostic purposes.In the latter instance, the CRISPR-Cas9 protocol defines with high degree of accuracy susceptibility to novel anti-virals, efficiently identifies molecular sequences correlated to virulence traits, and characterizes both the viral and host factors that determine resolution vs. chronicity of infections [23].

Animal studies have shown that CRISPR-based gene editing can also serve to introduce homology-directed repair into functional murine CD8+ T cells [24]. By inference, it follows that CRISP-Cas9 protocols may be developed to modify the epigenome and rescue exhausted T cells or recover Tregs [22] and control the cytokine storm [25] in SARS-CoV2 infected patients.It is possible and even probable that CRISP-Cas9 will be found to be as effective in blunting the pathology of inflammation at multiple stages, from loss of self-tolerance, to tissue damaged by inflammatory processes driven by cytokines, in CoVid-19 as reported in autoimmune diseases [26].

The natural history of the immunopathology of CoViD-19 might be outlined putatively in the following manner.

## Phase I:

Initial 5-7 days - monocyte/macrophage activation; pro-inflammatory cytokines unleash the 'cytokine storm' – It is possible and even probable that specific temporal phases characterize the release of IL1b, IL6, TNFa, TGFb and other cytokines, and that temporally controlled administration of specific monoclonal antibodies may counter these effects. It is also probable that the cytokine synthesis inhibitory factor (IL10) produced by Tregs plays a central and critical role at this stage. Flow cytometry of CD4, CD8, CD20 populations and of subpopulations are critical at this stage to better understand the unfolding of the immunopathology of CoViD-19, especially for the expression of activation markers such as CD71, the transferrin receptor that carries Fe into the activated T cell. Iron is essential for cell proliferation and activation. It is possible and even probable that impaired Fe availability, due to the effects of the virus on heme biology, alters CD71 expression by activated T cells, which in turn will decrease Tregs maturation, and progressively exhaust maturing cytotoxic T cells [22].

## Phase II:

Cytokine storms: It is possible and even probable that the cytokine storm is depleted in immunosuppressing IL-10. IL10-targeted immunotherapy at that stage of CoViD-19 may be beneficial 'to rescue' the patient by rescuing Tregs and reinstating functional cytotoxic CD8 from Tex.

In brief and in a simplified outline the CRISP-Cas9 protocol could entail starting with IL10 mRNA, the Tregs cytokine that blunts storming pro-inflammatory cytokines [22, 27,28]. By means of the protocol, that mRNA acts as guide mRNA, and reads the genetic information in host cell DNA until it finds the propitious spot for genomic editing. Cas9 locks at that site onto the double-stranded DNA, and unzips it to create a break in both strands of the DNA molecule to modulate the suppression of the IL10 gene and engender the recovery of the Treg function. The cell will repair the break by means of its endogenous mechanism. The protocol might reveal itself more challenging in practical terms. Nonetheless, this Prediction paper seeks not to proffer a clinical laboratory protocol, but rather a conceptual proof-of-principle.

## Discussion:

CoViD-19 is a complex disease that manifests a spectrum of signs and symptoms. The major clinical manifestations in Coronavirus infections, including SARS-CoV2 CoV are fever,confusion, chills, fatigue, generalized myalgia, malaise, drowsiness, cough, acute respiratory distress syndrome with dyspnea and pneumonia, severe gastrointestinal disorder with diarrhea, liver injury, coagulation dysfunction, and other signs and symptoms all derived from, or associated with a severe unregulated cytokine storm [29]. Abnormal coagulation parameter, leading to serous thrombi, are also found in patients with CoViD-19, which has led to the incorporation of anticoagulants, such as low molecular weight heparin, in treatment protocols [30]. Specifically, CoViD-19 patients with severe pneumonia exhibit significantly higher platelet count than patients with pneumonia not induced by SARS-CoV2; and only the former with markedly elevated D-dimer appear to benefit from anticoagulant treatment [31].

To be clear, the formation of thrombi is consequential to the activation of the coagulation cascade. Its intrinsic pathway is triggered in the context of a damaged blood vessel wall and exposure to collagen in the tissue space. Its extrinsic pathway initiates following activation of the serine protease proconvertin, factor VII, by CD142, platelet tissue factor, factor III (extrinsic pathway). Factor III, CD142, is, as a member of the cytokine receptor class II family, activated by cytokines. Type II cytokine receptors are transmembrane proteins that bind extracellularly and respond to a select group of cytokines. In the cytoplasmic region, type II cytokine receptors engage a tyrosine kinase of the Janus kinase family. Two species of type II cytokine receptors have been identified: those that bind type I and type II interferons, important activators of macrophages and inducers of autoinflammatory and autoimmune diseases; and those that bind the major anti-inflammatory cytokine IL10. The second species of type II cytokine receptors bind IL10 and IL20, a cytokine that regulates epidermal function and psoriasis, and which may in part responsible for the cutaneous manifestations in CoViD-19 [32], as well as IL22, which contributes to and enhances, via IL23 and related inflammatory cytokines, the 'cytokine storm'.

Both the intrinsic and the extrinsic pathways generate thrombin, factor IIa. It catalyzes the blood protein fibrinogen into fibrin, which aggregates into proteofibrils.Thrombin also activates the glycoprotein anti-hemophilic factor; factor XIII, which crosslinks the proteofibrils at the D fragment site, thus forming the fibrin scaffold for thrombus formation. One fibrin degradation product that results from plasmin-mediated fibrinolysis is the D-dimer, so-called because it contains two covalently linked D fragments. D-dimers are an important diagnostic tool diagnosis for intravascular coagulation and severe thrombotic disease [33], as are circulating pro-inflammatory cytokines [34].It follows that the plasmin system is another promising therapeutic target for combating CoViD-19 [35]. In addition to fever and generalized manifestations of the cytokine-mediated 'sickness behavior' [36], and to the noted respiratory illness and pulmonary pathology, liver and gastrointestinal tract disease, and skin lesions, infection with SARS-CoV2 leads to ocular abnormalities, from conjunctival hyperemia, chemosis, epiphora, or increased secretions in about one third of the more severe CoViD-19 patients [37]. Moreover, the high inflammatory burden from the SARS-CoV2 infection-induced 'cytokine storm' can lead to vascular inflammation, myocarditis, cardiac arrhythmias and related severe cardiovascular pathology [38]. Clinical evidence further supports SARS-CoV2 neuroinvasion [39]. This virus' neurotropic properties may provoke serious neurological diseases [40], including the reported loss of taste and smell [41]. Reports of mental dysfunction and depression in certain CoViD-19 patients are also emerging [42]. Taken together, these lines of evidence confirm the possibility of Neuro-CoViD-19 [43] as part of the natural history of SARS-CoV2 infection.

## Conclusions:

In conclusion, infection with SARS-CoV2 is responsible for CoViD-19, which manifests with an unusual high variety of symptoms, and multi-organ dysfunction, which can involve the gastrointestinal, cardiovascular, and respiratory systems, as well as specialized pathways, such as coagulation, and organs, including sight and of taste/smell, and central nervous functions. The condition displays a multi-dimensional spectrum of symptoms reminiscent more of a syndrome, than a specific disease. In conclusion, the natural history of this pathology may be better recognized as Corona Virus Syndrome 2019 (CoViS-19).
